# Influence of sociodemographic factors upon pain intensity in patients with temporomandibular joint disorders seen in the primary care setting

**DOI:** 10.4317/medoral.17576

**Published:** 2012-05-01

**Authors:** Antonio Blanco-Hungría, Alejandro Rodríguez-Torronteras, Antonio Blanco-Aguilera, Lourdes Biedma-Velázquez, Rafael Serrano-del-Rosal, Rafael Segura-Saint-Gerons, Javier de la Torre-de la Torre, Federico Esparza-Díaz

**Affiliations:** 1 MD, PhD, DDS. Andalusian Health Service. Collaborating Professor, Department of Medicine, Otorhinolaryngology and Dermatology. University of Córdoba; 2 MD, Epidemiologist. Córdoba Healthcare District, Andalusian Health Service; 3 DDS. MS University San Pablo-Ceu. Madrid; 4 Sociologist, MA. Senior Technical Research, CSIC. Institute for Advanced Social Studies (IESA-CSIC); 5 Sociologist, PhD. Research Scientist, CSIC. Institute for Advanced Social Studies (IESA-CSIC); 6 MD, PhD, DDS. Andalusian Health Service. Collaborating Professor, Department of Medical-Surgical Specialties. University of Córdoba; 7 MD, PhD, DDS, Andalusian Health Service

## Abstract

Objective: A study is made of the influence of gender, educational level, marital status, income, social support, and perceived general and oral health upon pain intensity in a sample of patients with temporomandibular joint disorders (TMJD) explored in primary care (AP). Design: A review was made of 899 patients from Córdoba Healthcare District (Spain) referred to the primary care TMJD Unit by their primary care physician and/or dentist. Of these subjects, 151 failed to meet the inclusion criteria. The remaining 748 subjects were explored according to the corresponding research diagnostic criteria (RDC/TMJD). A bivariate analysis was made the association of pain intensity to the demographic and psychological characteristics of the patients, and to perceived general and oral health, followed by a multivariate linear regression analysis to explain pain intensity as a function of the rest of the variables. The SPSS version 19.0 statistical package was used. Results: The patient age ranged from 18-86 years, with a mean of 45.8 years (± 15.8), and a female predominance of 5:1. The characteristic pain intensity (CPI) score was almost 15 points higher on average in women than in men (p<0.05). A lower educational level, and separation or divorce, were correlated to an increased intensity of pain. Social support, depression and general and oral health also explained part of pain intensity. The regression model established with these variables accounted for 13.3% of the variability of pain (R2 = 0.133). Conclusions: Women suffer more intense pain than men. Perceived health partially explains the variability of the CPI score. However, it is empirically seen that the variables gender, educational level and marital status exert an important and independent influence upon pain intensity.

** Key words:**Cranial-mandibular disorders, age, gender, educational level, primary care, research diagnostic criteria for temporomandibular joint disorders (RDC/TMD).

## Introduction

Temporomandibular joint disorders comprise a large group of signs and symptoms affecting the temporomandibular joints (TMJs), the muscles that control these joints, or both (1). The most commonly described symptom is localized or referred “pain” in the head and neck region.

Pain has precise physical, anatomical and pathological dimensions (2), but is also characterized by cultural or universal compo-nents in its expression and manifestation, with different interpretations from the social or cultural perspective (3). Although pain and its accompanying diseases have affected humans throughout history, it has not always been interpreted in the same way. This is due to the psychological, social and cultural dimensions of nociception, which make it necessary to investigate pain from both the physical and the social perspective – understanding pain as a socially measured component. In this sense, pain cannot be defined conceptually, since it is a somatic sensation that can only be known through the personal interpretation of the individual experiencing it (4).

In this context, pain is conditioned by the personal and particular elements of the individual suffering the condition, and also by the social elements that identify the individual, such as his or her sociodemographic characteristics. Accordingly, pain as a social concept implies the intervention of educational, cultural, sociological and personal elements, and as such requires study based on the subjective perception of the individual (5). In other words, if an individual with a lower educational level claims to suffer more pain than a person with a higher educational level, it may be because the former is suffering a more painful disorder. However, if in general and on a collective basis people with a lower educational level claim to suffer more pain than individuals with a higher educational level, we may suspect the existence of some influencing social component.

Therefore, in order to explore pain from a subjective perspective, we must take both social (age, gender, income, educational level, etc.) and psychological variables (depression, anxiety, self-esteem, affective relations, etc.) into account.

On the other hand, the research diagnostic criteria for temporomandibular joint disorders (RDC/TMJD) conform a dual-axis diagnostic classification system in which the first or clinical axis classifies the symptoms (using diagnostic algorithms) into one of the three most prevalent clinical presentations of TMJD (muscle disorders, disc disorders and joint disorders) (5,6). The second axis in turn measures the patient psychological condition (depression, physical symptoms related to anxiety and somatization), together with the intensity of pain and the degree of disability caused by it. The result is a classification of chronic pain to which are added a series of questions referred to mandibular function that define a degree of mandibular limitation (7,8). Application of the RDC/TMJD allows homogeneity or uniformity in registering the signs and symptoms of patients with TMJD, thereby ensuring greater precision in the study of this disease.

The prevalence of temporomandibular joint disorders has been extensively studied by many authors. However, on considering those that have used the RDC/TMJD (9-12), the prevalences are seen to vary, with pain reported by 80% among Israeli patients, muscle disorders in 38% of Italian patients, and joint disorders in 50% of Asian patients. This important variability in the signs and symptoms is also noted in the demographic parameters such as gender, with a markedly higher percentage of pain associated to TMJD in women (ranging from 2.6 women with pain per male in the Italian group, to 3.5 women per male in the Israeli group).

Authors such as LeReshe the al. (13) have explored the influence of hormones, and particularly of the estrogens, as an etiological factor in the appearance and perpetuation of orofacial pain and temporomandibular joint disorders (14). Fillingim et al. (15) placed special emphasis on the biopsychosocial factor as being responsible for the increased pain in women. In turn, authors such as Greenspan et al. (16), through the publication of a consensus document, have facilitated future research lines allowing us to better understand the origin of such differences.

The rest of sociodemographic variables such as educational level, marital status, or income have been little investigated in relation to TMJD (17).

The present study explores the relationship between patient perceived pain intensity and the sociodemographic variables gender, age, educational level, marital status and income, variables pertaining to the psychological domain, and self-reported general and oral health.

## Material and Methods

The study sample consisted of 899 patients referred between January 2007 and July 2010 by primary care physicians and dentists ascribed to Córdoba Healthcare District (Spain). The following inclusion criteria were established: age: ≥18 years (since the RDC/TMJD has not been used or validated in individuals under age 18 years), with some of the following signs or symptoms: mandibular or TMJ pain, limitation or restriction during oral aperture or lateralization, or joint sounds with or without pain.

The following exclusion criteria were applied: systemic rheumatological (except fibromyalgia or rheumatoid arthritis), neurological or autoimmune diseases, patients subjected to TMJ surgery or head and neck irradiation treatment, patients with head and neck injuries in the two months prior to the study, pregnant women, the use of psychoactive medication, muscle relaxants or corticosteroids not suspended at least one week before the study, antidepressant or nonsteroidal antiinflammatory drug use within the past three days, patients with antecedents of drug abuse, and subjects failing to give written informed consent to participation in the study.

Of the original 899 patients, we excluded two who were under 18 years of age, while 149 declined to participate in the study. These subjects did not differ from the remaining 748 patients in terms of the demographic variables. The patients who agreed to participate returned for a second visit in which the two axes of the RDC/TMJD were evaluated.

Patient data compilation and the clinical examination were carried out according to the RDC/TMJD guidelines, using a validated translated version ensuring intra- and inter-examiner reproducibility (18,19).

The study questionnaire addressed sociodemographic variables, psychological variables, clinical parameters and pain intensity. Regarding the sociodemographic variables, the main characteristics of the patient were considered: gender, age, educational level, occupational status at the time (employed or otherwise), marital status and income. In turn, two psychological variables were assessed: satisfaction with the social support received (persons from which help can be received in case of need), and degree of depression. The latter was rated according to the modified Symptoms Checklist-90 (SCL 90)(20,21), comprising 20 items (wanting to cry, feelings of guilt, loneliness, sadness, etc.). The resulting overall score is the sum of the scores corresponding to each item, divided by the total number of variables, and ranges from 0 (no symptoms of depression) to 4 (extreme depression) as a continuous numerical scale.

**Figure 1:**
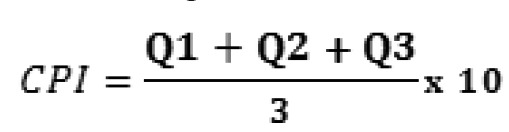
Formula to obtain the pain intensity (CPI).

The characteristic pain intensity (CPI) score was obtained from the GPS (Global Pain Scale), applying the following formula: (Fig. 1) 

where Q1 is the intensity of pain at the present time; Q2 is the worst pain intensity of the last 6 months; and Q3 is the average pain intensity of the last 6 months – based on a scale of 0 (no pain) to 10 (worst pain possible). The result of the sum is divided by the number of questions, multiplied by 10. In this sense, the result is a new variable with values from 0 (no pain) to 10 (intense pain).

Lastly, patient perceived general and oral health was explored by means of a 5-point Likert scale.

A univariate descriptive analysis was made of the different study variables. In turn, the influence of the sociodemographic, psychological and health variables upon pain intensity among the patients with temporomandibular joint disorders in the primary care setting was evaluated based on a bivariate analysis (contrasting of means using the Snedecor F-statistic) of each of the variables and pain intensity.

After assessing the bivariate relationships between the CPI score and rest of the variables, we developed a linear regression model in which the mentioned score was taken to be the dependent variable, while the independent variables were those parameters found to show a significant association to the CPI score in the bivariate analysis.

The SPSS version 19.0 statistical package was used throughout. The study was approved by the Ethics Committee of Reina Sofía University Hospital (Córdoba, Spain).

## Results

The main descriptors of the CPI score are shown in  Table 1. In turn, as can be seen from  Table 2, the sociodemographic, psychological and health characteristics of the patients showed a statistically significant association to the intensity of pain - with the exception of income and occupation.

**Table 1:**
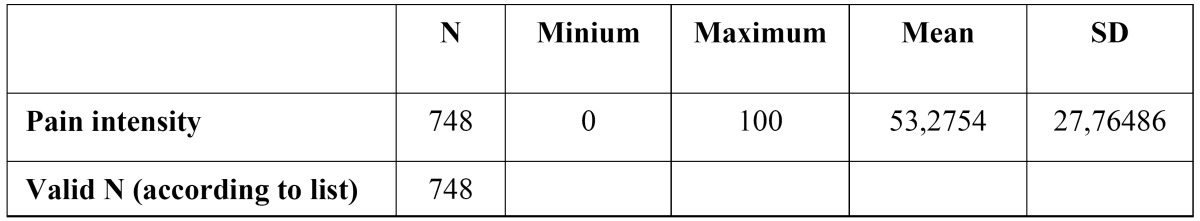
Descriptive statistics relatiing to the characteristic pain intensity (CPI) scores.

**Table 2:**
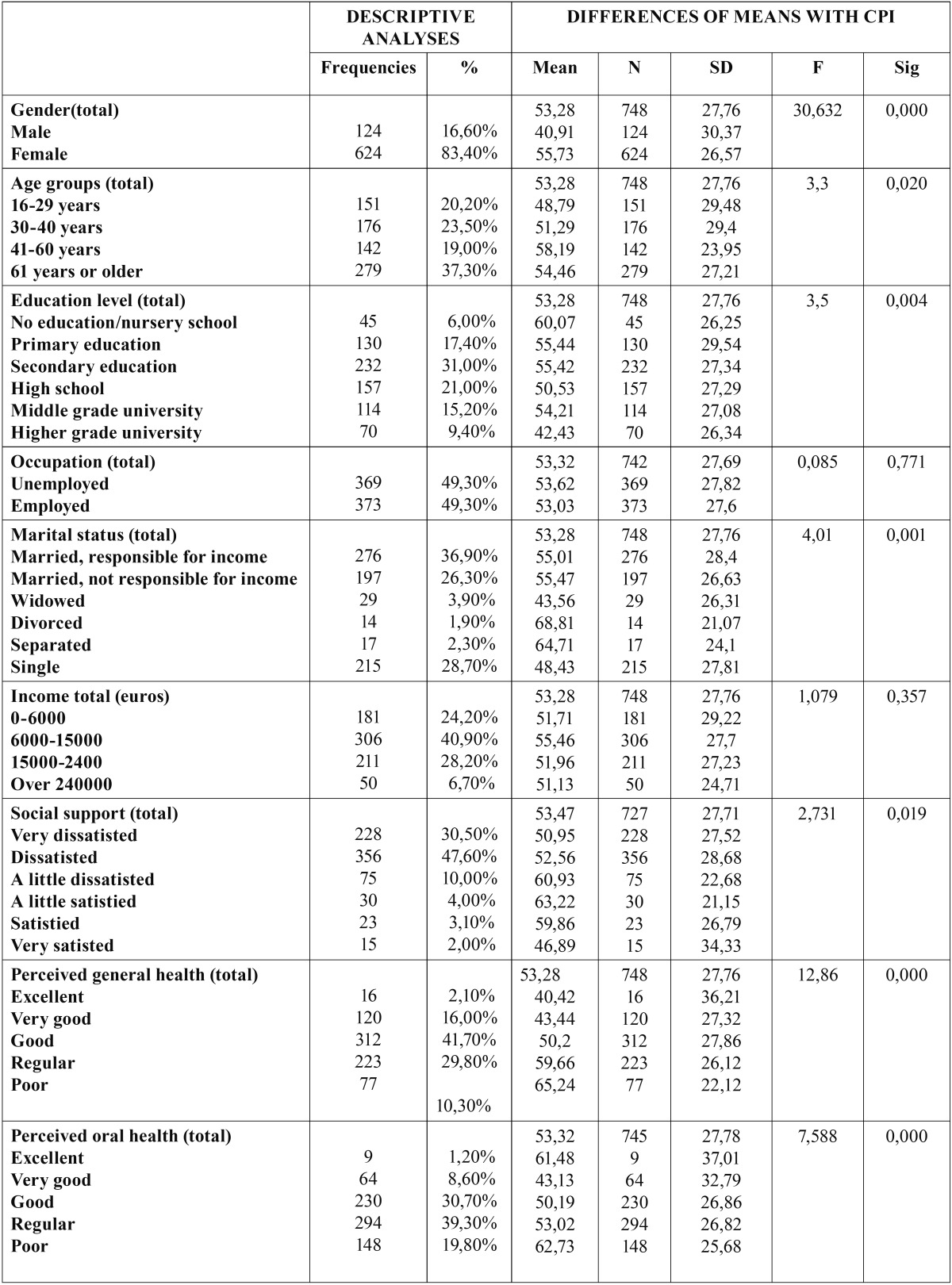
Descriptive analysis of the sociodemographic, psychological and perceived health variables, and differences of means with the characteristic pain intensity (CPI) scores.

**Figure 2:**
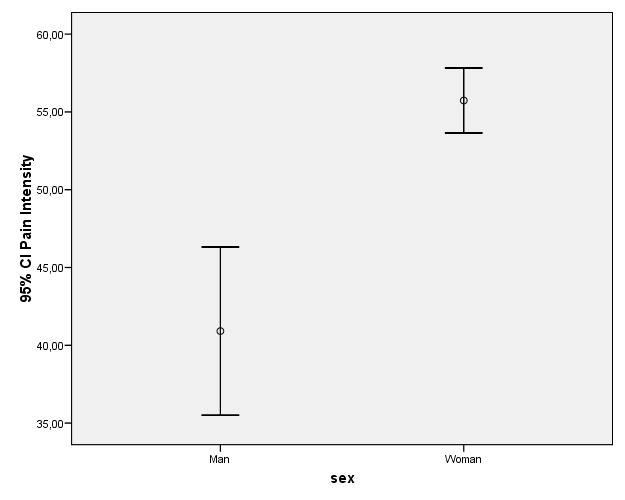
Differences in CPI score and 95% confidence interval according to patient gender.

The characteristic pain intensity (CPI) score was almost 15 points higher on average in women than in men (55.73 and 40.91 respectively). As can be seen from figure 2, the difference is statistically significant.

Another significant element in the bivariate analysis with respect to CPI was educational level. Those patients with a lesser educational level yielded higher CPI scores, while those with a higher education level yielded comparatively lower pain scores.

**Figure 3:**
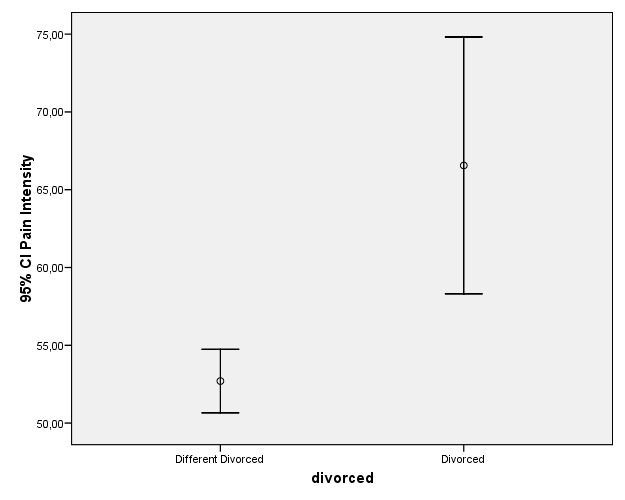
Differences in CPI score and 95% confidence interval according to patient marital status (separated / divorced or rest of categories).

Regarding marital status, separated or divorced patients reported higher intensity pain, followed by married subjects, while single or widowed patients yielded the lowest pain scores. The differences between the separated or divorced patients and the rest of the marital status categories proved significant (Fig. 3).

A higher or lower income, or being employed or not, implied no changes in CPI score.

In reference to the psychological variables, the availability of social support was significantly correlated to the CPI score. In general, the patients were clearly dissatisfied with the social support received. While differences were observed, they were mainly circumscribed to those individuals clearly satisfied with the support received (satisfied or very satisfied) versus the rest of the patients – the latter exhibiting the highest CPI scores ( Table 2).

On the other hand, a positive association was observed between depression and an increased intensity of pain.

Lastly, regarding the two health variables, higher CPI scores were associated to poorer perceived health, as could be expected.

The results of the regression analysis are reported in  Table 3. Three regression studies were made: the first included only the sociodemographic variables, the second also contemplated the psychological variables, and the third furthermore included the perceived health variables. Each of these regression analyses are detailed below:

**Table 3:**
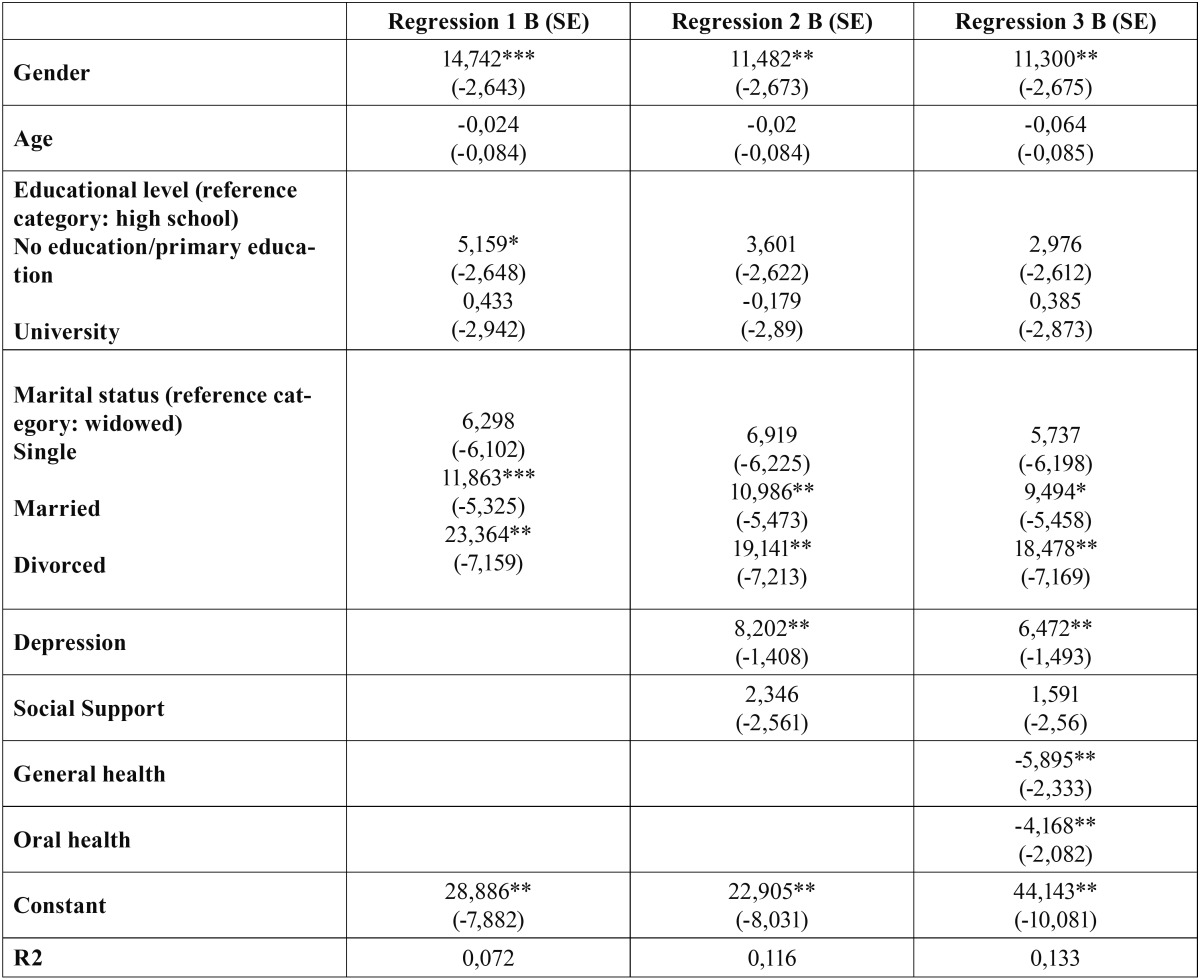
Descriptive analysis. Dependent variable: CPI score. Independent variables: Regression 1 (includes only sociodemographic variables); Regression 2 (includes sociodemographic and psychological variables); Regression 3 (includes sociodemographic, psychological and health variables).

Regression 1: The variables found to be significant were gender, married or divorced status (confidence level 95%), and primary education or no education (confidence level 90%). The variables found to be significant in the bivariate but which lost significance in the regression analysis were age, university studies and single status. The model proved globally significant (F=8.180). The determination coefficient (R2) was not very high and accounted for 7.2% of the pain intensity. Considering that the analysis focused on the effect of the sociodemographic variables upon pain, this should not be interpreted as a low value. The coefficients (B) indicate the variation in pain intensity in response to unit variation in some of the variables, assuming that the rest of the variables remain constant. According to the model, gender increment (0 representing males and 1 females) raised the pain intensity score by 14.7 points. The same was seen to apply to educational level, where only primary education or no education implied a pain intensity increase of over 5 points versus patients with higher educational levels. Lastly, married or divorced status implied a pain intensity increment of 11.8 and 23.3 points, respectively.

Regression 2: This analysis furthermore included the variables of the psychological and social support axis of the RDC/TMJD. As a result, the R2 of the model increased 4.4%, and the global independent variables accounted for 11.6% of the variability in patient pain intensity. The variables found to be significant were gender, married or divorced status, and depression (confidence level 95%). Educational level, which proved significant in the previous model, was no longer significant in this second regression analysis. This may be due to the observation that the subjects with a lower educational level also showed the highest depression and lack of social support scores. Likewise, while the bivariate analysis revealed significance for the variable social support, this significance was lost in the regression study. The model proved globally significant (F=10.484). The coefficients (B) were similar to those of the previous analysis, with some differences in terms of the increment observed in the dependent variable. Regarding depression, the patient pain intensity score was seen to increase 8 points for each increment in depression (0 representing no depression and 4 indicating extreme symptoms of depression).

Regression 3: Lastly, this third regression analysis included the above variables together with the two parameters related to patient perceived health. These variables were introduced in order to determine their effect upon the rest, allowing us to evaluate the influence of the sociodemographic and psychological variables upon pain intensity after inclusion in the model, independently of the effects of patient health proper. It is very interesting to note that there were no relevant changes in the determination coefficients of the previously included variables. The model proved globally significant (F=9.919), explaining 13.3% of the variability in pain intensity. An increase in perceived general and oral health was seen to be associated with a 5- and 4-point decrease in pain intensity (negative coefficients), respectively.

## Discussion

The present study shows that women suffer more intense pain than men, in coincidence with the observations of other studies. According to Rohlfs et al. (22), the health of women and men is different and uneven: different because men and women do not suffer the same illnesses as a result of biological factors, or do so with different intensity and risk; and uneven because there are social factors that influence health, and which are conceived differently according to gender. An example of this can be observed on analyzing how depression influences pain intensity; however, women moreover suffer greater degrees of depression than men, as a result of which the effects multiply.

According to Bonjardin (23), there is a clear imbalance between men and women, though this investigator failed to observe significant differences for some parameters of TMJD, such as for example occlusal factors (occlusion and Angle class). Nevertheless, the difference proved significant for anxiety, though not for depression. In contrast to the above, Mundt et al. (24) not only recorded greater involvement among females but also identified the loss of occlusal support as a significant factor in the development of TMJD.

However, Pereira et al. (25) reported that increased disability, depression and somatization may be diagnostic indicators of TMJD, particularly in relation to pain intensity (CPI), with an up to 31-fold greater influence in adolescent females than in men. In the same way as in our study, on comparing with pain intensity (CPI), a correlation was observed with the sociodemographic variables gender and marital status.

An interesting observation in our study is that patient perceived health explained part of the variability of the CPI score, but did modify the effects of the sociodemographic and psychological variables included in the model when health was omitted. In other words, the effect of gender, educational level or marital status upon pain intensity is independent of perceived health.

We did not observe a statistically significant relationship between pain intensity and age group, occupation at the time of the study (employed or otherwise), or income.

As explained by Martins et al. (17), the observation that people with a lower educational level experience more pain than people with a higher educational level cannot be accounted for in terms of biological factors; rather, the explanation is to be found in the global effects of the underlying social influences. In order to counter this situation, it is necessary to identify those elements of greatest relevance in explaining the social inequalities in relation to health, as corroborated by our own observation that pain intensity is greater in patients with a low educational level than in those with higher level education.

In conclusion, while we have found that there are biological elements that condition differences in patient health, it remains important to study the social and cultural differences responsible for certain concrete aspects of such differences. In this context, the primary care setting, as the first step in the provision of health care, offers an excellent scenario for the conduction of such research.
